# A test of the diffusion model explanation for the worst performance rule using preregistration and blinding

**DOI:** 10.3758/s13414-017-1304-y

**Published:** 2017-02-23

**Authors:** Gilles Dutilh, Joachim Vandekerckhove, Alexander Ly, Dora Matzke, Andreas Pedroni, Renato Frey, Jörg Rieskamp, Eric-Jan Wagenmakers

**Affiliations:** 1 Department of Psychology, University of Basel, Missionsstrasse 60/62, 4055 Basel, Switzerland; 2 University of California Irvine, Irvine, CA USA; 3 University of Amsterdam, Amsterdam, The Netherlands

**Keywords:** Response time model, Preregistration, Predictions, Intelligence

## Abstract

People with higher IQ scores also tend to perform better on elementary cognitive-perceptual tasks, such as deciding quickly whether an arrow points to the left or the right Jensen (2006). The worst performance rule (WPR) finesses this relation by stating that the association between IQ and elementary-task performance is most pronounced when this performance is summarized by people’s slowest responses. Previous research has shown that the WPR can be accounted for in the Ratcliff diffusion model by assuming that the same ability parameter—drift rate—mediates performance in both elementary tasks and higher-level cognitive tasks. Here we aim to test four qualitative predictions concerning the WPR and its diffusion model explanation in terms of drift rate. In the first stage, the diffusion model was fit to data from 916 participants completing a perceptual two-choice task; crucially, the fitting happened after randomly shuffling the key variable, i.e., each participant’s score on a working memory capacity test. In the second stage, after all modeling decisions were made, the key variable was unshuffled and the adequacy of the predictions was evaluated by means of confirmatory Bayesian hypothesis tests. By temporarily withholding the mapping of the key predictor, we retain flexibility for proper modeling of the data (e.g., outlier exclusion) while preventing biases from unduly influencing the results. Our results provide evidence against the WPR and suggest that it may be less robust and less ubiquitous than is commonly believed.

Over the past decades, the field of mental chronometry has revealed several robust associations between high-level cognitive ability (e.g., IQ, working memory) and response times (RT) in elementary cognitive-perceptual tasks (Jensen 2006; Van Ravenzwaaij et al. 2011). The main finding is that people with relatively high IQ-scores tend to respond relatively quickly in simple RT tasks that do not appear to involve deep cognitive processing; one example of such a task is the random dot kinematogram, which requires participants to detect the direction of apparent motion in a cloud of dot stimuli.

Another important finding is known as the worst performance rule (WPR): the fact that the worst performance in these simple tasks—that is, the slowest responses—is most indicative of high-level cognitive ability (Baumeister and Kellas 1968; Larson and Alderton 1990). In this study, we aimed to assess the presence and intensity of the WPR in a large data set. In addition, we test a prediction from the Ratcliff diffusion model (Ratcliff 1978; Ratcliff et al. 2008), namely that speed of information processing is the factor that underlies the WPR.

In order to ensure that our statistical assessment is fair (e.g., unaffected by hindsight bias or confirmation bias), we first preregistered our entire analysis plan and submitted it to *Attention, Perception, & Psychophysics* (e.g., Chambers, 2013; Wolfe, 2013). Only after approval by the journal did we start to analyze the data. The preregistration plan can be found online at https://osf.io/qc5dh/.

A novel element to our preregistration proposal is the inclusion of a blinding procedure, where an analyst (in this case, author JV) is sent the data with the key variable shuffled (MacCoun and Perlmutter 2015). This way, the analyst is free to (1) resolve ambiguities and oversights in the preregistration document; and (2) adjust the analysis to unexpected peculiarities of the data. Crucially, this freedom of analysis does not endanger the confirmatory nature of the statistical inference: shuffling the key variable breaks the analysis-outcome feedback loop that compromises the confirmatory status of the inference. Only after the analyst had committed to the analysis plan was the key variable unshuffled.

## The worst performance rule

**Fig. 1:**
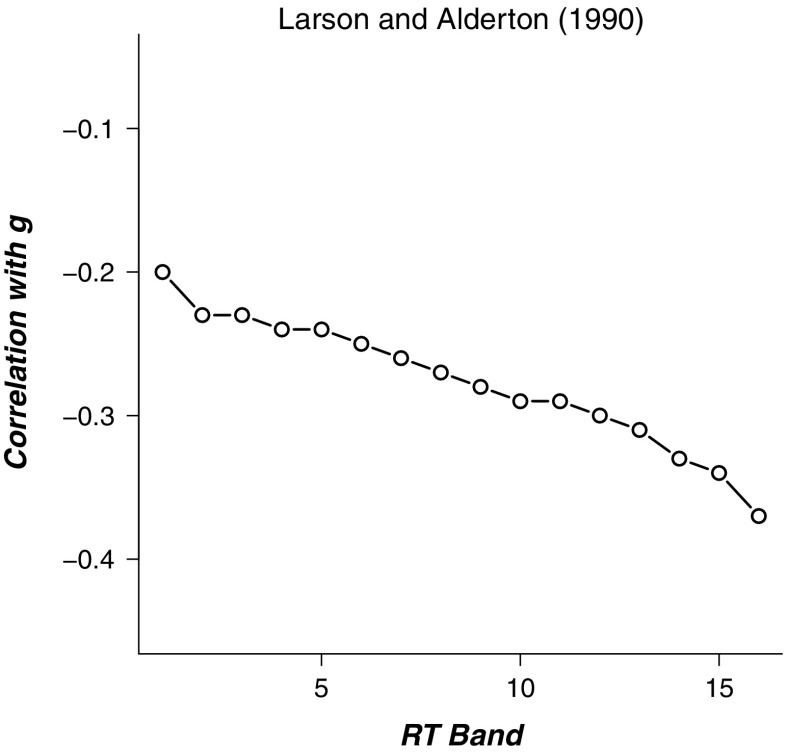
An example of the worst performance rule. The negative correlation of RT with IQ gets stronger as RTs lengthen. Data from Larson and Alderton (1990)

Since the seminal work by Baumeister and Kellas (1968), the WPR has been shown to exert itself in various forms. In its most general form, the WPR holds that the worst performance on multi-trial elementary cognitive-perceptual tasks is more predictive for *g-loaded* measures than is the best performance on these tasks (Coyle 2003). This prediction is usually confirmed by demonstrating that higher RT bands correlate more strongly than lower RT bands with both IQ measures (e.g., Larson & Alderton, 1990; Jensen, 1982) and working memory capacity (WMC; e.g., Unsworth, Redick, Lakey, & Young, 2010). For example, Fig. 1 presents the results from Larson and Alderton (1990), showing that the negative correlation between RT and IQ gets stronger as RT lengthens.

The WPR expresses itself in several related ways as well. Coyle (2001), for example, found that the worst performance on a word-recall task (i.e., the lowest number of words from a list recalled by each participant) correlates higher with IQ than the best performance on this task (i.e., the highest number of words from a list recalled by each participant). Furthermore, Kranzler (1992) and Ratcliff et al. (2010) showed that the WPR is strongest for multi-trial tasks that are relatively complex.

Several explanations for the WPR have been proposed. The most dominant explanation holds that performance on cognitive tasks of any level in any domain (e.g., IQ, WMC, speeded perceptual choice) is facilitated by the general neural processing speed of an individual’s brain (Jensen 2006). Inspired by this idea, Ratcliff et al. (2008) suggested that the drift rate parameter of the diffusion model reflects precisely this speed of processing.

## The Ratcliff diffusion model

**Fig. 2:**
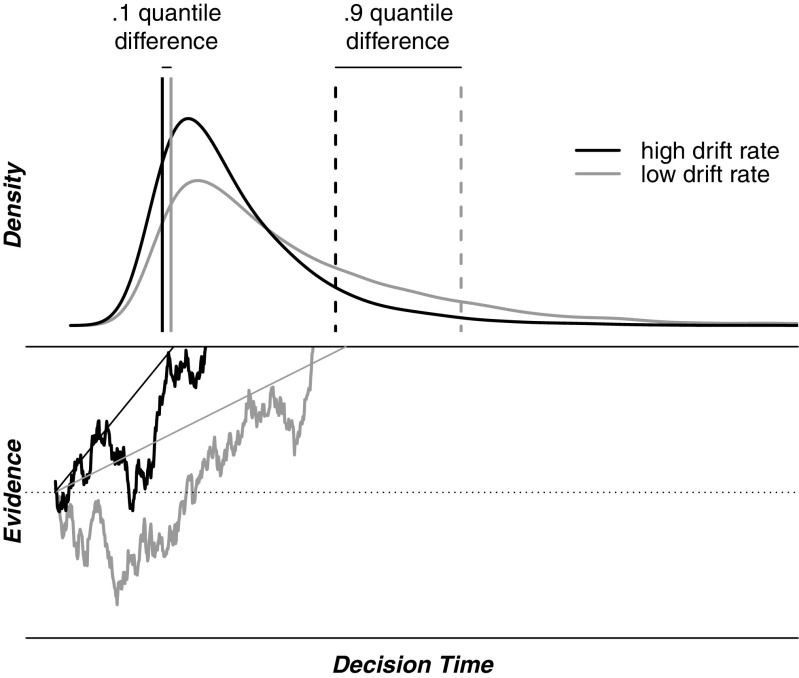
The Ratcliff diffusion model. Noisy evidence is accumulated until one of two pre-set boundaries is reached. The *lower* half of the figure shows two exemplary accumulation paths (*meandering lines*) and two different drift rates (the average rate of information accumulation, *straight lines*). The *upper part* shows the correct RT distributions that result from a low and a high drift rate. *Vertical lines* indicate the shift in .1^*st*^ (*solid lines*) and .9^*th*^ (*dashed lines*) percentiles caused by a change in drift rate

The diffusion model (Ratcliff 1978) describes the observed RT distributions of correct and error responses on two-choice tasks as the finishing times of a diffusion process with absorbing bounds. When presented with a stimulus, a decision-maker is assumed to accumulate noisy evidence from that stimulus (i.e., the meandering lines in Fig. 2) until either of two pre-set evidence boundaries is reached and the associated response is initiated. On average, the accumulation of evidence approaches the correct boundary at a speed that is quantified by the drift rate parameter. Due to noise in the accumulated evidence, the diffusion process sometimes reaches the incorrect boundary, leading to error responses. This within-trial noise is also responsible for the right-skewed distribution of RT. In the model’s most extended form, the diffusion process is governed by seven parameters, including drift rate. Thus, drift rate is a key parameter of the diffusion model, as it corresponds to the signal-to-noise ratio in the evidence accumulation process; hence, drift rate quantifies the speed of information processing.

Ratcliff et al. (2008) pointed out an important property of the diffusion model for the explanation of the WPR: increasing drift rate acts to reduce RT. Crucially, this reduction is most pronounced for higher percentiles of RT (cf. Van Ravenzwaaij et al., 2011), as is illustrated in the upper part of Fig. 2. The figure shows RT distributions that originate from two different drift rates. The solid vertical lines indicate the .1 quantiles of the distributions resulting from a high drift rate (dark line) and a low drift rate (grey line). The dashed vertical lines indicate the .9 quantiles of these distributions. Clearly, the change in drift rate leads to a larger shift of the slow .9 quantile than of the fast .1 quantile. Thus, differences in drift rate and differences in IQ have the same qualitative effect on RT, in the sense that both are most strongly expressed in the slowest RTs. This observation adds credibility to the idea that the diffusion model’s drift rate parameter quantifies the speed of processing that is thought to underlie the WPR as well as other associations between higher-level and lower-level cognitive tasks. In order to test this idea, several empirical studies related drift rate to IQ and WMC. Ratcliff et al. (2010) and (2011) showed that IQ correlated positively with drift rate in recognition memory tasks. Ratcliff et al. (2010) further showed that IQ correlated positively with drift rate in a lexical decision task and a numerosity judgment task. A study by Leite (2009), however, found no evidence of a correlation between IQ and drift rate in either a brightness discrimination task or a letter discrimination task. Schmiedek et al. (2007) showed that WMC could be predicted from drift rate on a range of RT tasks.^1^

**Fig. 3:**
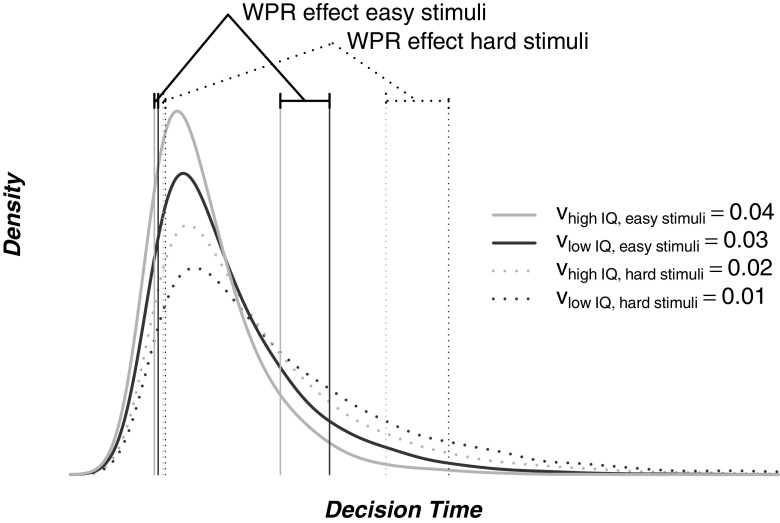
Four hypothetical drift rates *v* for easy stimuli (*solid lines*) and difficult stimuli (*dotted lines*), for participants with a relatively high IQ (*light lines*) and participants with a relatively low IQ (*dark lines*). The density lines show the predictions of the diffusion model, given these drift rates. The vertically drawn quantile lines show that the IQ effect on the higher ranges of RT (i.e., the .9 quantile) relative to the lower range of RT (i.e., the .1 quantile) is stronger for the difficult than for the easy stimuli

Another important observation about the relation of drift rate and RT was made by Van Ravenzwaaij et al. (2011). The diffusion model holds that both stimulus difficulty and subject ability are expressed in drift rate. In fact, drift rate can be viewed as a pair of scales weighting two intrinsically related constructs: difficulty and ability. The drift rate is the deflection of the pointer of this scale and is most pronounced in the slowest RTs, that is, in the worst performance. From this observation, Van Ravenzwaaij et al. (2011) suggested that difficulty, just as ability (e.g., IQ), should be reflected most strongly in the higher ranges of RT, a prediction that was empirically confirmed by Van Ravenzwaaij et al. (2011). From this same interconnection of IQ and difficulty, we hypothesize that the WPR is more pronounced for difficult than for easy items of an elementary RT task. Figure 3 illustrates this hypothesis with a concrete example. The figure shows four hypothetical correct RT distributions generated by four drift rates that differ across IQ group and stimulus difficulty. The effect of IQ on slow (.9 quantile) responses is larger than the effect on fast (.1 quantile) responses. This difference is more pronounced for difficult stimuli (dotted lines) than for easy stimuli (solid lines). This prediction is closely in line with the observations of Kranzler (1992) and Ratcliff et al. (2010), who showed that more complex tasks show a more pronounced WPR.

## Overview of hypotheses

The current study presents a rigorous, preregistered test of four hypotheses related to the WPR and the account provided by the Ratcliff diffusion model. First, we test the existence of the WPR. Second, we test the prediction that the WPR is larger for difficult than for easy trials in a simple RT task. Third, we test the prediction that the diffusion model drift rate parameter correlates with WMC. Fourth, we test the prediction that the correlation between drift rate and WMC is higher for difficult trials than for easy trials from the perceptual RT task. We test these hypotheses by analyzing an existing data set with 916 participants for which we measured both perceptual choice RT and WMC. A detailed account of the design, hypothesis, and proposed analyses is provided below.

## Data collection and method

The data at hand have been collected in a large-scale study on the genetic underpinnings of risk preferences, funded by the Swiss National Science Foundation. For this study, 916 participants (502 participants in Berlin, Germany; 414 in Basel, Switzerland) were tested on a range of psychological tasks. Among the participants, 65 *%* were students, and 62 *%* were female. The age range spans 18-36 years with a mode at 24 years. For the current study, we analyze the data of two relevant tasks: a WMC test and a perceptual two-choice RT task.

### Working memory capacity battery

To measure working memory, we used the WMC battery developed by Lewandowsky et al. (2010). This battery was constructed as a tool to measure working memory capacity with a heterogeneous set of tasks that involves both verbal and spatial working memory. A pre-defined measurement model described in Lewandowsky et al. (2010) allows the calculation of a single WMC score for each participant. Lewandowsky et al. (2010) show that this score has a strong internal consistency and correlates highly with Raven’s test of fluid intelligence ( *r*=.67).

### Speeded perceptual two-choice task

**Fig. 4:**
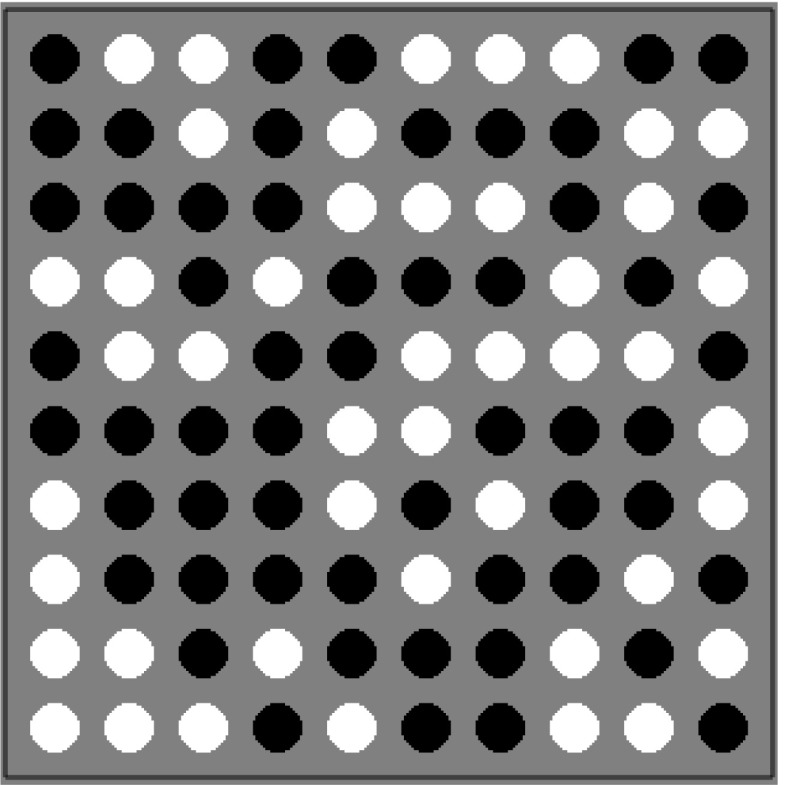
Example of a stimulus in the perceptual RT task. Participants pressed the *left* or *right* mouse button to indicate quickly whether the stimulus contained more *black* or more *white* dots

In the elementary RT task, participants were presented with 10×10 matrices of black and white dots (Fig. 4). Participants were instructed to indicate whether the matrix contained more black or more white dots by pressing either of two mouse buttons. In this simple perceptual task, difficulty can be manipulated by adjusting the number of black and white dots. Participants saw 90 easy trials (proportion of black and white dots: 60/40, 40/60) and 90 difficult trials (proportions 55/45, 45/55). In addition, there were trials with an equal proportion of black and white dots. These stimuli are “undoable”, and are of no special interest in this perceptual task but were included for comparison with another task conducted in the large-scale study. In the current analyses, we nonetheless include these trials in order to facilitate the estimation of the diffusion model parameters. Participants received no feedback, but were instructed to respond as fast and accurately as possible. A “too slow” message was displayed after responses slower than 3.5 s. Our task originates from Dutilh and Rieskamp (2016) and resembles tasks that have been modeled successfully with the diffusion model, such as the brightness discrimination task (Ratcliff and Rouder 1998) and the numerosity task (Ratcliff et al. 2010).

## Registered analysis plan

In this study, our goal was to test four key hypotheses in a manner that is described in detail below. For all hypotheses, we use the Bayes factor to quantify the degree of confirmation provided by the data (Jeffreys 1961); we will also provide the posterior distribution for the parameters of interest.

The registered analysis plan was carried out on the complete data set (subject to the outcome-blind decisions by the analyst; see the next section on the two-stage analysis process). In a second, exploratory analysis, we will test the hypotheses separately for the relatively homogeneous student group and the relatively heterogeneous non-student group.

Note that, with 1000 participants, we collected data that are sufficiently informative to pass Berkson’s “interocular traumatic test” (Edwards et al. 1963) such that the confirmatory hypothesis tests serve merely to corroborate what is immediate apparent from a cursory visual inspection of the data. Below, we provide a description of the hypotheses and analyses that is consistent with the original preregistration plan; as will become apparent later, the analyst executed some outcome-independent changes to this original plan.

### Planned analysis of hypothesis 1: Worst performance rule

For each participant, we obtained a single WMC score from the WMC battery. Furthermore, for each participant we obtained the 1/6, 2/6, 3/6, 4/6, and 5/6 quantiles of correct RTs; it is possible to use more quantiles, but only at the cost of reducing the precision with which the mean RT within each bin is estimated. Hypothesis 1 states that the correlation between WMC and mean RT within each quantile is negative (i.e., higher WMC is associated with faster responding). More specifically, Hypothesis 1 states that the absolute magnitude of this correlation increases monotonically from the fastest to the slowest quantile (i.e., the WPR). Hypothesis 1a refers to the WPR for easy stimuli, and Hypothesis 1b refers to the WPR for difficult stimuli.

Both Hypothesis 1a and 1b are tested separately, in the following manner. Denote by *ρ*_*i*_ the estimated Pearson correlation coefficient for quantile *i*. Then, the simplest linear version of the WPR predicts that *ρ*_*i*_ = *β*_0_ + *β*_1_ *I*_*i*_, where *I*_*i*_ indicates the quantile, *β*_0_ is the intercept of the regression equation, and *β*_1_ is the slope. We then use the Bayes factor (Jeffreys 1961; Kass and Raftery 1995) to quantify the support that the data provide for two competing hypotheses: the null hypothesis $\phantom {\dot {i}\!}\mathcal {H}_{0}: \beta _{1} = 0$ versus the WPR alternative hypothesis $\phantom {\dot {i}\!}\mathcal {H}_{1}: \beta _{1} < 0$. Under $\phantom {\dot {i}\!}\mathcal {H}_{1}$, we assign each *ρ*_*i*_ an independent uniform prior from −1 to 0, in order to respect the fact that all correlations are predicted to be negative. Furthermore, we assign a uniform prior to *β*_0_ that ranges from −1 to 0, in order to respect the fact that even for the fastest RTs, the correlation is not expected to be positive. Finally, we assign a uniform prior to *β*_1_ that ranges from its steepest possible value to 0. Specifically, since the quantiles are on the scale from zero to one, and the highest possible value of the intercept *β*_0_ equals 0, the assumption of linearity across the scale implies that the steepest slope is −1. Hence, we assign *β*_1_ a uniform prior from −1 to 0 (see the results section for an inconsistency in this model specification).

With the model specification in place, the Bayes factor between $\phantom {\dot {i}\!}\mathcal {H}_{0}\!\!\!:\!\!\!\! \beta _{1}\!\!\!\!\!\! =\!\!\!\!\! 0$ versus $\phantom {\dot {i}\!}\mathcal {H}_{1}\!\!\!\!: \beta _{1}\!\!\!\!\ \sim \!U[-1,0]$ can be obtained using an identity known as the Savage–Dickey density ratio (e.g., Dickey & Lientz, 1970; Wagenmakers, Lodewyckx, Kuriyal, & Grasman, 2010). Specifically, this involves focusing on parameter *β*_1_ in $\phantom {\dot {i}\!}\mathcal {H}_{1}$ and comparing the prior ordinate at *β*_1_=0 to the posterior ordinate at *β*_1_=0, that is, by computing $\phantom {\dot {i}\!}\text {BF}_{10} = p(\beta _{1}=0 \mid \mathcal {H}_{1})/p(\beta _{1}=0 \mid y, \mathcal {H}_{1})$, where *y* denotes the observed data. Bayes factors higher than 1 favor $\phantom {\dot {i}\!}\mathcal {H}_{1}$ and provide support for the WPR. All parameters will be estimated simultaneously using a hierarchical Bayesian framework and Markov chain Monte Carlo (MCMC, e.g., Lee & Wagenmakers, 2013).

### Planned analysis of hypothesis 2: Stronger worst performance rule for more difficult stimuli

The WPR tested under Hypothesis 1 is predicted to be more pronounced for difficult stimuli than for easy stimuli. In the previous WPR model, *ρ*_*i*_ = *β*_0_ + *β*_1_ *I*_*i*_; now denote *β*_1_ for the difficult stimuli by *β*_1*d*_ and denote *β*_1_ for the easy stimuli by *β*_1*e*_. Hypothesis 2 holds that *β*_1*e*_>*β*_1*d*_. We multiply both parameters by −1 so that we obtain variables on the probability scale, and hence $\phantom {\dot {i}\!}\beta ^{*}_{1d}>\beta ^{*}_{1e}$. We use a dependent prior structure (Howard 1998), apply a probit transformation, and orthogonalize the parameter space (Kass and Vaidyanathan 1992). Specifically, denoting the probit transformation by Φ^−1^, we write $\phantom {\dot {i}\!}{\Phi }^{-1}(\beta ^{*}_{1d}) = \mu + \delta /2$ and $\phantom {\dot {i}\!}{\Phi }^{-1}(\beta ^{*}_{1e}) = \mu - \delta /2$. We assign the probitized grand mean parameter *μ* an uninformative distribution, that is, *μ*∼*N*(0,1), and then use the Bayes factor to contrast two models: the null hypothesis $\phantom {\dot {i}\!}\mathcal {H}_{0}: \delta =0$ versus the alternative hypothesis $\phantom {\dot {i}\!}\mathcal {H}_{2}: \delta > 0$. We complete the model specification for $\phantom {\dot {i}\!}\mathcal {H}_{2}$ by assigning the difference parameter *δ* a default folded normal prior defined only for positive values, that is, *δ*∼*N*(0,1)^+^. As before, parameter estimates are obtained from MCMC sampling in a hierarchical Bayesian model and Bayes factors will be computed using the Savage–Dickey density ratio test on parameter *δ* under $\phantom {\dot {i}\!}\mathcal {H}_{2}$.

### Planned analysis of hypothesis 3: Working memory capacity correlates positively with drift rate

We fit the diffusion model to the data using hierarchical Bayesian estimation (e.g., Wabersich & Vandekerckhove, 2014; Wiecki, Sofer, & Frank, 2013). This hierarchical method allows us to exploit the vast number of participants and estimate parameters even for participants whose data contain little information (for example due to a small number of errors, which are crucial for diffusion model parameter estimation). Hypothesis 3 holds that WMC correlates positively with drift rate. Hypothesis 3a refers to the positive correlation between WMC and drift rate for the easy stimuli, and Hypothesis 3b refers to the positive correlation between WMC and drift rate for the difficult stimuli. Both Hypothesis 3a and 3b will be tested separately, in the following manner.

First WMC is included within the hierarchical structure. WMC will then be correlated with drift rate estimates (Hypothesis 3a: for the easy stimuli; Hypothesis 3b: for the difficult stimuli) in a hierarchical structure. The null hypothesis holds that there is no correlation, $\phantom {\dot {i}\!}\mathcal {H}_{0}: \rho = 0$, whereas the alternative hypothesis holds that the correlation is positive, $\phantom {\dot {i}\!}\mathcal {H}_{3}: \rho > 0$. Specifically, we assign *ρ* a uniform prior from 0 to 1. Bayes factors can be obtained by a Savage–Dickey density ratio test on parameter *ρ* under $\phantom {\dot {i}\!}\mathcal {H}_{3}$.

### Planned analysis of hypothesis 4: Stronger correlation between working memory and drift rate for more difficult stimuli

Hypothesis 4 holds that WMC correlates more strongly with drift rates for difficult stimuli than with drift rates for easy stimuli. Denote by *ρ*_*d*_ the WMC-drift rate correlation for the difficult stimuli, and by *ρ*_*e*_ the WMC-drift rate correlation for the easy stimuli. Hypothesis 4 states that *ρ*_*d*_>*ρ*_*e*_. Moreover, both *ρ*_*d*_ and *ρ*_*e*_ are assumed to be positive, so that both are on the probability scale. Consequently, the proposed analysis mimics that of Hypothesis 2: We use a dependent prior structure, apply a probit transformation, and orthogonalize the parameter space. We write Φ^−1^(*ρ*_*d*_) = *μ* + *δ*/2 and Φ^−1^(*ρ*_*e*_) = *μ*−*δ*/2. We assign the probitized grand mean parameter *μ* an uninformative distribution, that is, *μ*∼*N*(0,1), and then use the Bayes factor to contrast two models: the null hypothesis $\phantom {\dot {i}\!}\mathcal {H}_{0}: \delta =0$ versus the alternative hypothesis $\phantom {\dot {i}\!}\mathcal {H}_{4}: \delta > 0$. We complete the model specification for $\phantom {\dot {i}\!}\mathcal {H}_{4}$ by assigning the difference parameter *δ* a default folded normal prior defined only for positive values, that is, *δ*∼*N*(0,1)^+^. As before, parameter estimates are obtained from MCMC sampling in a hierarchical Bayesian model and Bayes factors will be computed using the Savage–Dickey density ratio test on parameter *δ* under $\phantom {\dot {i}\!}\mathcal {H}_{4}$.

## Two-stage analysis

We pursue an unbiased yet flexible method to test the diffusion model account of the WPR. Therefore, we adopted a two-stage analysis with a special status for coauthor JV who fits the diffusion model to data (e.g., Vandekerckhove & Tuerlinckx, 2007, 2008; Vandekerckhove, Tuerlinckx, & Lee, 2011; Wabersich & Vandekerckhove, 2014). In the first stage, we provided JV with the perceptual RT data and a *randomly permuted* version of the WMC variable. With these data in hand, JV produced code to fit the model while respecting the analysis choices outlined above (i.e., Hypotheses 1–4). This first stage allowed JV to model the data at will, for instance by excluding outliers, introducing contaminant processes, adding transformations, and generally make any other reasonable modeling choice. Importantly, JV was also able to correct ambiguities and oversights in the preregistration document that had initially escaped us. Since the crucial WMC score variable is randomly permuted, the correlation between drift rate and WMC estimated in this stage-one model is meaningless. The first stage was terminated when JV indicated the model code is ready. At this point, the code was fixed and made available on the Open Science Framework (https://osf.io/wupbm). In the second stage, the true sequence of WMC scores was revealed, and the code created by JV was applied to the data in a deterministic manner to address each of the hypotheses outlined above.

This two-stage analysis is both flexible and fair. It is flexible because the modeler retains the freedom to exclude data and make adjustments to the model to account for eventual peculiarities of the data, and it is fair because the modeling choices are not outcome-driven, that is, guided by expectations about the main hypotheses.

## Results of preregistered analyses

After the methods and analysis plan above were preregistered and accepted as such at *Attention, Perception & Psychophysics* on October 1^*st*^, 2015, author JV prepared the preregistered analyses based on the blinded data. For hypotheses 1 and 2, JV had to deviate slightly from the analysis plan. This deviation solved an inconsistency in the original analysis description. Thanks to the fact that the analyst was blinded, the findings of this analysis remain purely confirmatory.

On May 11, 2016, JV registered the analysis plan on the Open Science Framework (https://osf.io/wupbm), at which moment the unblinded data set was shared with JV. After lifting the blind, a small typo was found in the analyses codes for both Hypotheses 3b and 4. This typo involved the coding of stimulus types (left, right, hard, easy). The nature of this typo and its correction are unambiguous, and we believe that the analyses can still be considered purely confirmatory. The model code for all analyses is available at https://osf.io/qc5dh/.

### Descriptive results

**Fig. 5:**
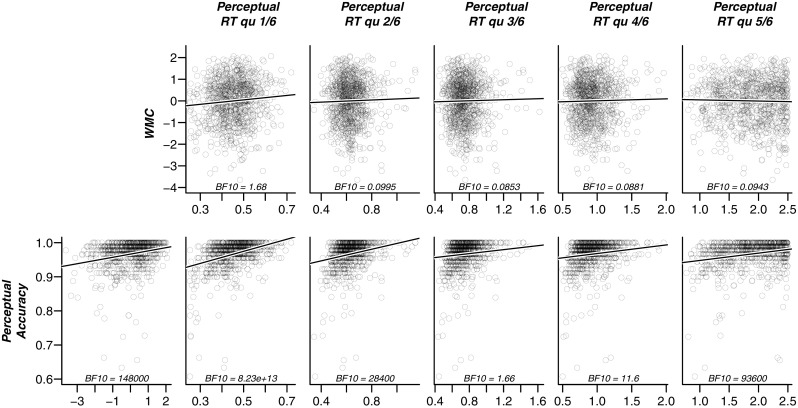
Scatterplots of key outcome variables for the easy items. *Upper row*: relation between WMC and each of the five quantiles (.1, .3, .5, .7, .9) of correct RT; *Lower row*: relation between accuracy and each of the five quantiles of correct RT. The first panel in the lower row shows the relation between overall accuracy and WMC. Each *point* represents a participant. Each *panel* shows the Bayes factor in favor of a linear model with non-zero slope (represented by the *black line*) versus the intercept-only model. Bayes factors are calculated from the BayesFactor Package for R (Morey et al. 2014)

Before we turn to the results of our preregistered analyses, we first present a descriptive view of the observed data in Figs. 5 (easy items) and 6 (hard items) to facilitate the understanding of our results. In these figures, individual participants are presented by points, and the lines illustrate linear regressions fitted to these points. In both figures, the panels in the upper row show the relation between working memory capacity versus each of the five quantiles of correct RT. In the lower row, the first panel shows the relation between working memory capacity and overall accuracy. The remaining five panels in the lower row display the relation between overall accuracy and each of the five quantiles of correct RT.

**Fig. 6:**
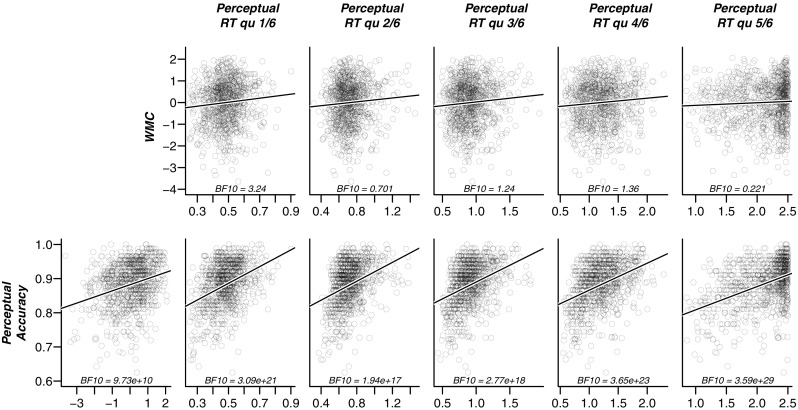
Scatterplots of key outcome variables for the hard items. *Upper row*: relation between WMC and each of the five quantiles (.1, .3, .5, .7, .9) of correct RT; *Lower row*: relation between accuracy and each of the five quantiles of correct RT. The first panel in the lower row shows the relation between overall accuracy and WMC. Each *point* represents a participant. Each *panel* shows the Bayes factor in favor of a linear model with non-zero slope (represented by the *black line*) versus the intercept-only model. Bayes factors calculated from the BayesFactor Package for R (Morey et al. 2014)

These figures support a number of observations. First, for both easy and hard items, the left-most panel of the lower row reveals a clear positive correlation between WMC and accuracy; in other words, participants with high WMC are relatively accurate on the perceptual task. Second, although all our hypotheses predict a *negative* correlation between WMC and RT, the panels in the upper row suggest that in our sample of participants, such a relation is absent. For the fastest RTs, there is even weak evidence for a positive relation. Finally, the five right-most panels in the lower row highlight that accuracy correlates positively with RT. In other words, participants who respond slowly also respond more accurately. We keep these observations in mind when we present the results of the confirmatory hypothesis tests below.

### Hypotheses 1a and 1b

Hypothesis 1 states that working memory capacity correlates negatively with mean RT in the bins defined by the 1/6, 2/6, 3/6, 4/6, and 5/6 quantiles of correct RT, both for easy and hard stimuli. The precise prediction that we tested was that the negative correlation increases linearly over the RT bins. When working on the blinded data, an inconsistency was discovered in the preregistered analysis plan for this hypothesis. This analysis plan specified both the slope parameter *β*_1_ and the correlations *ρ*_*i*_ of WMC with each quantile of RT as estimable parameters. Since *ρ*_*i*_ is defined as a function of *β*_1_, only one of both can be estimated. This inconsistency was corrected while working on the blinded data and only the beta weight was defined as an estimable parameter.

**Fig. 7:**
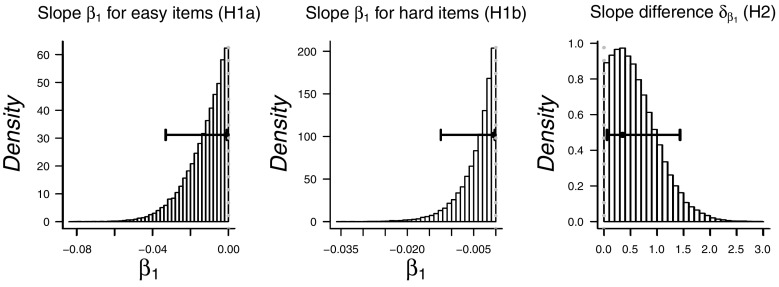
Posterior distributions for parameters *β*_1_ for easy and hard items, as estimated under Hypotheses 1a and 1b and for difference parameter *δ*, as estimated under Hypothesis 2. *Whiskers* indicate 95 *%* highest density intervals

The results show strong evidence against Hypothesis 1a that stipulates a negative *β*_1_ for the easy items ( BF_01_=64.3) and strong evidence against Hypothesis 1b that stipulates a negative *β*_1_ for the hard items ( BF_01_=222). This support for a zero *β*_1_ constitutes evidence *against* the WPR in its classical form. This result is not too surprising given the apparent absence of negative a negative relation observation that we observed in Figs. 5 and 6. Indeed, when we present the exploratory results, it will become apparent that the reason for the strong support against Hypotheses 1a and 1b is that, when estimated freely, the correlations between RT and WMC are actually slightly positive rather than negative, such that people who respond more slowly tend to have larger WMC. This positive rather than negative correlation is also suggested by the posterior distributions of *β*_1_ for easy and hard items in Fig. 7, which show most of their mass at the 0-edge of the prior parameter range.

**Fig. 8:**
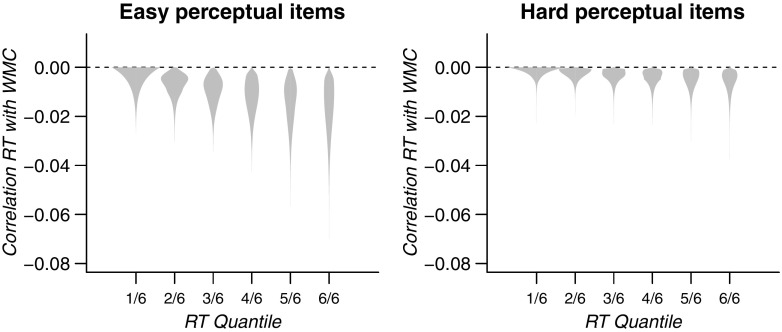
Correlation between RT quantiles and WMC, as defined from the linear function estimated in confirmatory analyses H1a and H1b. The densities reflect the uncertainty about these correlations that follow from the uncertainty about the parameters of the linear function. Note that no statistical evidence was found for a negative slope

For illustration of the results of this analysis, Fig. 8 shows the correlations between RT quantiles and WMC as defined by the linear function that was estimated in the model. The densities indicate the uncertainty of each correlation following from the uncertainty in the estimate of the parameters of the linear function.

### Hypothesis 2

Hypothesis 2 states that the linear decrease tested under hypothesis 1b (hard stimuli) was stronger than the decrease tested under hypothesis 1a (easy stimuli). Although we found evidence against the existence of each of these WPR effects, we can still test whether one effect is stronger than the other. The Bayes factor indicates inconclusive evidence about this hypothesis ( BF_01_=1.10). The posterior distribution of the difference parameter *δ* is displayed in the rightmost panel of Fig. 7.

### Hypotheses 3a and 3b

**Fig. 9:**
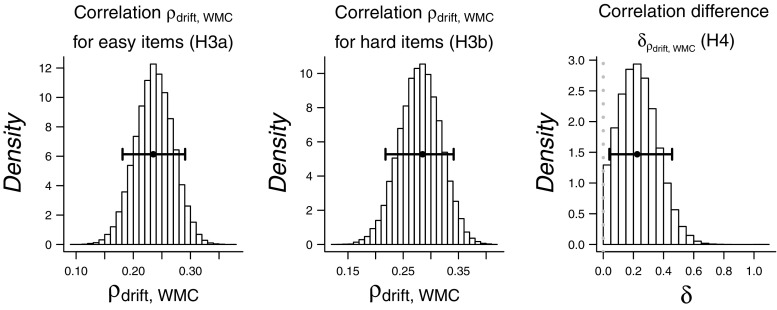
Posterior distributions for correlations *ρ* between drift rate and WMC for easy and hard items, as estimated under Hypotheses 3a and 3b and for difference parameter *δ*, as estimated under Hypothesis 4. *Whiskers* indicate 95 *%* highest density intervals

Hypothesis 3 states that working memory capacity correlates positively with the diffusion model drift rate on the perceptual task, for both easy (hypothesis 3a) and hard (3b) stimuli. Both hypotheses 3a and 3b are confirmed with strong evidence (hypothesis 3a: BF_10_=58.7, hypothesis 3b: BF_10_=889). The estimated correlations with working memory capacity were 0.24 for easy and 0.28 for hard stimuli. The posterior distributions of the correlations are displayed in the leftmost panels of Fig. 9.

### Hypothesis 4

Hypothesis 4 states that the correlation between working memory and drift rates is higher for hard than for easy stimuli. The Bayes factor shows inconclusive evidence about this hypothesis ( BF_01_=1.25). The posterior distribution of the difference parameter *δ* is displayed in the rightmost panel of Fig. 9.

## Discussion of preregistered hypotheses

In its standard form, the worst performance rule predicts that the correlation between working memory capacity and perceptual RT is negative, and that this negative correlation becomes increasingly pronounced for the higher RT bands. Our preregistered analysis revealed strong evidence against the worst performance rule, for both easy and hard items (i.e., Hypotheses 1a and 1b).

Interestingly, however, we did find strong evidence for a positive correlation between the perceptual drift rates and working memory capacity (i.e., Hypotheses 3a and 3b). This finding supports the conceptual idea underlying the WPR: higher-level processing and lower-level processing are facilitated by the same general processing speed. The evidence for a difference in the strength of this effect between easy and hard items was inconclusive.

Initially, these two main results may seem inconsistent; after all, the hypothesis of a correlation between working memory and drift rate was raised since this correlation could produce the worst performance rule in its classical form. Therefore, it is remarkable that we found strong evidence *in favor* of a correlation between perceptual drift rate and working memory capacity, but comparably strong evidence *against* the classical WPR hypothesis. The exploratory analyses below aim to address this issue.

## Results of exploratory analyses

Inspection of the descriptive data in Figs. 5 and 6 suggest two explanations for our seemingly conflicting set of results, that is, evidence against a negative correlation between WMC and RT, and evidence in favor of a positive correlation between WMC and perceptual drift rate. The first explanation is that we have put inappropriate constraints on the correlations of WMC with RT: these correlations were constrained to be negative, although in the data they appear slightly positive. This misspecification may be responsible for our failure to find the classical WPR.

The second explanation is that the drift rate is a more specific measure of general processing speed than response times. The diffusion model explicitly describes how response times can be influenced by factors other than the speed of processing, such as the caution with which decisions are made. Thus, large individual differences in response caution across participants might have masked the worst performance rule in its classical form. Both explanations will be discussed and tested below.

### Explanation 1: Undue constraints on correlations

Our preregistered hypotheses specified all correlations between RT and WMC to be negative: people with higher WMC were expected to respond more quickly on the perceptual task, an effect expected to increase over RT bands. Therefore, in the statistical analyses for Hypotheses 1 and 2, these correlations were restricted to fall between –1 and 0. Figures 5 and 6 suggest that this prediction may be incorrect: if anything, the correlations appear to be positive. The correlations may in fact appear to decrease monotonically over quantiles. It is possible, therefore, that this pattern is masked by the constraint that all estimated correlations should be negative. The analysis below examines whether it was this misspecification that kept us from detecting a true worst performance rule in the data.

#### Results releasing constraints on correlations for Hypotheses 1a, 1b, and 2

**Fig. 10:**
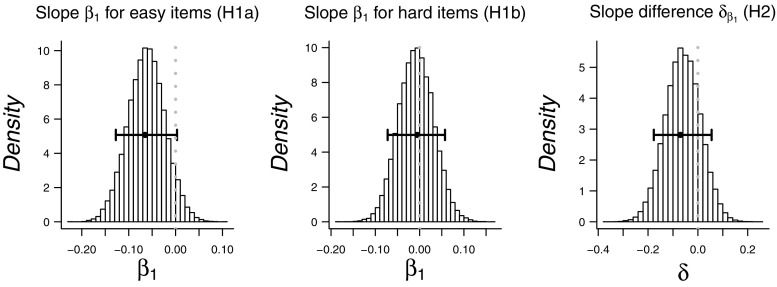
Exploratory analysis: Posterior distributions for parameters *β*_1_ for easy and hard items and for difference parameter *δ*, as estimated when releasing the constraints on the correlations for Hypotheses 1a and 1b, and 2. *Whiskers* indicate 95 *%* highest density intervals

**Fig. 11:**
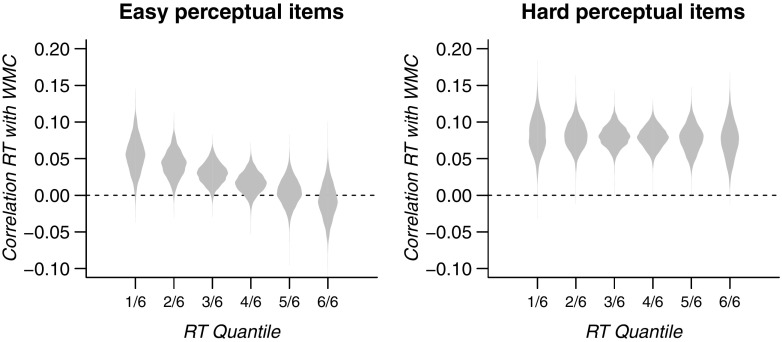
Exploratory analysis: Correlation between RT quantiles and WMC, as defined from the linear function estimated in confirmatory analyses H1a and H1b. The densities reflect the uncertainty about these correlations that follow from the uncertainty about the parameters of the linear function. Note that no statistical evidence was found for a negative slope

In a revision of analyses 1a, 1b, and 2, we release the constraint on correlations to be strictly negative; specifically, we release the constraints on the intercept and slope of the linear function relating quantile number to the correlations. After releasing these constraints, the intercept ( *β*_0_) was indeed estimated to be positive for both easy and hard items of the perceptual task. At the same time, the slope ( *β*_1_) was estimated to be slightly negative, as illustrated by the posterior distributions of the *β*_1_ parameters depicted in the leftmost panels of Fig. 10. For the easy perceptual items, these *β*_0_ and *β*_1_ values defined a linear function with positive WMC–RT correlations for all but the highest quantile of perceptual RT. For the hard perceptual items, all correlations defined by this linear function are positive. These linear functions are illustrated in Fig. 11. Again, the black dots and grey line indicate the highest density estimate of this linear function, whereas the densities depict the uncertainty around the resulting individual correlations.

Although these estimates suggest that releasing the constraint on the correlation function improved the analyses, we again found evidence opposing the worst performance rule hypothesis for both easy stimuli (Hypothesis 1a^*^, *B* *F*_01_=6.29) and hard stimuli (Hypothesis 1b^*^, *B* *F*_01_=19.9). Thus, releasing the constraint on the correlations did not result in a different conclusion and the evidence still favors the absence of the worst performance rule in its classical form, albeit less strongly than for the restricted analysis.

### Explanation 2: Confound of response caution

A possible theoretical explanation for the fact that we find evidence against the WPR in its classical form is that true WPR effects are confounded by individual differences in response caution. To understand this explanation, consider the scenario in which participants who are more cautious than others on the perceptual task, also more carefully perform the WMC test. Careful participants will score higher on the WMC test and respond more slowly (and maybe more accurately) on the perceptual task. The result could be a *positive* correlation between WMC and perceptual RT. More precisely, the diffusion model predicts that an increased boundary separation on both tasks will result in a positive correlation between WMC and RT that increases over RT bands (Ratcliff et al. 2008), an effect that opposes the WPR predictions.

This observation offers an alternative interpretation for the results in Figs. 5 and 6: On the one hand, there exists a true correlation between WMC and drift rate; a correlation that is assumed under the WPR and found in our confirmatory analyses, which would, unopposed, cause a *negative* correlation between RT and WMC in the form of the worst performance rule. Acting against this influence, however, are individual differences in boundary separation and carefulness that cause a *positive* correlation between RT and WMC. Below, we study this alternative interpretation in more detail.

#### Results correcting for potential confound

To test whether individual differences in response caution have confounded the worst performance rule on the raw RT data, we explore how analyses 1a, 1b, and 2 turn out when we perform them on ten subgroups with similar response caution. These ten homogeneous–caution groups were created by dividing participants based on the .1, .2, .3, .4, .5, .6, .7, .8, .9 quantiles of the boundary separation estimates obtained in analysis 4. The results of this analysis did, however, not yield consistent results either. Only in one boundary separation bin (the .3 through .4 quantile), and then again only for easy stimuli, there appeared to be a decrease of the correlation over quantiles. Thus, accounting for the potential confound of response caution does not alter our conclusions about the existence of the WPR on the raw RT data.

### Student vs. non-student participants

In the preregistration document, we foreshadowed exploratory analyses to study whether the worst performance rule would show more reliable for the relatively homogeneous student-sample, than for the rest of the participants. For this exploratory analyses, we repeated the worst-performance hypothesis tests H1a, H1b, and H2 separately for students (*n* = 690) and non-students (*n* = 211, for 15 participants, there was no information available as to whether they were students or not). As was the case in the full data set, the analyses for neither the students sub-sample, nor the non-students sub-sample, yielded noteworthy evidence in favor of the WPR hypothesis. These results are available in the online appendix on OSF (https://osf.io/7dwfy/).

## General discussion

### Summary of results

We tested four main hypotheses to study the worst performance rule. The first two hypotheses concerned the relationship between working-memory capacity and perceptual choice response times. Hypothesis 1, which formalized the classical WPR hypothesis predicting a negative correlation between working-memory capacity and all quantiles of perceptual RT that strengthens over the quantiles of RT, was rejected for both easy and hard perceptual stimuli (hypotheses 1a and 1b). Even after releasing the order-constraints on the correlations in our exploratory analyses, the evidence spoke against the worst performance rule in its classical form. We also explored the possibility that individual differences in response caution had confounded a true latent WPR. A separate analysis of groups of participants with homogeneous response caution did not lead to different conclusions. Given these results, it is not surprising that the related Hypothesis 2, that the WPR would hold more strongly for hard than for easy items, was not supported.

The second set of hypotheses concerned the explanation of the worst performance rule in terms of the diffusion model’s drift rate. Hypothesis 3 formalized this explanation by predicting a positive correlation between working memory capacity and the diffusion model drift rate in the perceptual task. This hypothesis was strongly confirmed for both easy and hard perceptual stimuli. However, no evidence was found for Hypothesis 4 that stated that working memory capacity correlates stronger with drift rates for hard than for easy perceptual stimuli.

### Interpretation of results

Our results suggest that the worst performance rule is more fragile than the literature suggests. Whereas many studies have reported support for the worst performance rule, our preregistered analyses revealed evidence for the absence of the effect. It is also of note that the size of the correlations between WMC and drift rate are moderate in comparison to those found in similar studies (e.g., Schmiedek et al., 2007; Ratcliff et al., 2010, 2011; Schmitz & Wilhelm, 2016). Our preregistered results were obtained in a large data set and, to a skeptical by-stander, it may appear that the ubiquity and robustness of the worst performance rule results in part from selective reporting and publication bias. This worrying possibility can only be excluded by additional large-scale preregistered studies.

### Preregistration and blinding

This study reported the first purely confirmatory and unbiased test of the well-studied worst performance rule. To achieve this goal, we preregistered an analysis plan that was conditionally accepted for publication in *Attention, Perception, & Psychophysics* (Wolfe 2013). We anticipated that the required modeling effort would be relatively complex, and therefore we incorporated a blinding protocol in which the analyst (author JV) developed the analysis code based on a version of the data set in which the crucial WMC variable was shuffled. This shuffling prevented JV to alter the analysis plan in order to achieve desirable outcomes. Our personal experience with the blinding protocol was highly positive, as it secured fairness without sacrificing flexibility.

### Conclusions

Our results show strong evidence for the claim that the same underlying processing speed, as quantified by the diffusion model drift rate, underlies perceptual choice and working memory capacity. Thereby our results support the theoretical explanation of the worst performance rule. The worst performance rule itself, however, was absent in our data. These results raise the question of how ubiquitous the worst performance rule really is, a question that can only be addressed by additional studies using preregistration and blinding.

## Footnotes

1
^1^ In fact, Schmiedek et al. (2007) constructed a measurement model to distill for each participant a latent factor for drift rate, boundary separation, and non-decision time.
